# Development of a tRNA-Derived Small RNA Prognostic Panel and Their Potential Functions in Osteosarcoma

**DOI:** 10.3389/fonc.2021.652040

**Published:** 2021-08-02

**Authors:** Zhenming Tang, Shuhui Zhang, Zhougui Ling

**Affiliations:** Department of Pulmonary and Critical Care Medicine, The Fourth Affiliated Hospital of Guangxi Medical University, Liuzhou, China

**Keywords:** osteosarcoma, tsRNA, miRNA-seq, prognostic panel, survival

## Abstract

**Background:**

Therapeutic outcomes of osteosarcoma treatment have not significantly improved in several decades. Therefore, strong prognostic biomarkers are urgently needed.

**Methods:**

We first extracted the tRNA-derived small RNA (tsRNA) expression profiles of osteosarcoma from the GEO database. Then, we performed a unique module analysis and use the LASSO-Cox model to select survival-associated tsRNAs. Model effectiveness was further verified using an independent validation dataset. Target genes with selected tsRNAs were predicted using RNAhybrid.

**Results:**

A LASSO-Cox model was established to select six prognostic tsRNA biomarkers: tRF-33-6SXMSL73VL4YDN, tRF-32-6SXMSL73VL4YK, tRF-32-M1M3WD8S746D2, tRF-35-RPM830MMUKLY5Z, tRF-33-K768WP9N1EWJDW, and tRF-32-MIF91SS2P46I3. We developed a prognostic panel for osteosarcoma patients concerning their overall survival by high-low risk. Patients with a low-risk profile had improved survival rates in training and validation dataset.

**Conclusions:**

The suggested prognostic panel can be utilized as a reliable biomarker to predict osteosarcoma patient survival rates.

## Introduction

Osteosarcoma is one of the most common primary malignant tumors in bone development from the mesenchymal cell line and occurs mostly in adolescents with a worldwide incidence of 3.4/1,000,000 per year (1). Rapid tumor growth is due to the direct or indirect form of tumor osteoid and bone tissue through the cartilage stage (2). The disease puts a heavy burden on children and their parents and brings both irreparable psychological trauma and financial strain to patients’ families and society. Although a combination of neoadjuvant chemotherapy and extensive surgical resection somewhat improves patient outcomes, the overall 5-year disease-free survival rate is only about 65% (3). The traditional prognostic factors for osteosarcoma patients are broad, including tumor size, location, grade, metastasis, and sensitivity to chemotherapy. This diversity of prognostic factors prevents a direct comparison between the criteria. Since metastasis and sensitivity to chemotherapy are important prognostic indicators of osteosarcoma, they have been criticized on multiple grounds. In the last three decades, there has been no improvement in the survival of osteosarcoma patients. Thus, it is of great importance to identify new early-stage diagnostic biomarkers and novel therapeutic targets for early diagnosis and quantitative assessment of osteosarcoma prognosis to improve patient survival. tRNA derived small RNA (tsRNA) is a kind of new small non-coding RNA, usually 18 to 40 nucleotides in length, produced by the precursor tRNA or mature tRNA (4). tsRNA can be divided into three different categories, including precursor tRNA, derived small RNA with poly-U residue characteristic of 3’ terminal (3’U tRF), Mature tRNA derived fragment (tRF), and tRNA half molecules (tRH). tRF can be further divided into three subtypes: 5’tRF 3’tRF and Inter tRF. The biogenesis of different tsRNAs is regulated by different mechanisms. For instance, during the maturation of tRNA, 3’U tRF is produced by RNase Z, and angiogenin cleavage of anticodon rings of mature tRNA generates tRHs, indicating that tsRNA expression is regulated by space and time under physiological conditions, playing a significant role in many biological processes (5). Moreover, tsRNAs can regulate mRNA translation, retro-element reverse transcriptional, and post-transcriptional processes (6). Hence, the function of tsRNA is likely to be elucidated by examining its interactions with mRNA. A growing number of studies show the importance of aberrant tsRNA expression during cancer development and staging resulting from activation of oncogenes and inactivation of tumor suppressors (7). The potential importance of tsRNAs as non-invasive diagnostic factors, prognostic biomarkers, and therapeutic targets has been seriously considered.

## Methods

### Dataset

The miRNA-seq dataset collected from (Massachusetts General Hospital) is available in the SRA repository (SRP237494). The mRNA dataset was obtained from the TARGET-OS project (dbGap phs000468). When data were separated into training and validation sets, the training to validation ratio was 7:3. Independent validation patient samples were collected from the Fourth Affiliated Hospital of Guangxi Medical University.

### Clinical Samples

This study was a prognostic cohort test approved by the Fourth Affiliated Hospital of Guangxi Medical University’s ethics committee. Blood samples were collected from 30 patients (**Table 1**), which included patients with OS in our hospital from January 2011 to March 2020. Informed written consent was obtained from each patient. Blood samples were centrifuged at 3,000 g for 15 minutes at 4°C and then stored at -80°C.

**Table 1:** Clinical characteristics of osteosarcoma.

Cohort	MGH	TARGET	Independent Validation
**Num. of patients**	74	88	30
**Age in years, mean (SD)**	29.39 (0.23)	15.17 (0.06)	28.16 (0.55)
**Sex**			
Male, count (%)	49 (66.2)	51 (58.0)	11 (36.7)
**Metastases(%)**			
No	57 (77)	Not Reported	22 (73.3)
Yes	17 (23)		8 (26.7)
**Chemotherapy regime**			
MAP or AP	29	Not Reported	Not Reported
MAP and other	40		
Other	3		
Not available	2		
**Chemo-response**			
Optimal	25	Not Reported	Not Reported
Suboptimal	26		
Not available	23		
**Events**			
Death	28	29	11
Recurrences	36	Not Reported	15
**Follow Up time (moths), mean (SD)**	98.2 (0.92)	50.8 (0.43)	96.16 (2.07)

### Reverse Transcription-Quantitative PCR (RT-qPCR)

According to the manufacturer’s protocol, total RNA from OS blood was extracted using TRIzol reagent (Invitrogen; Thermo Fisher Scientific, Inc.) Consequently, complementary DNA (cDNA) was synthesized using the QuantiTect Reverse Transcription Kit (QIAGEN, Inc.) Relative expression qPCR was conducted on StepOne™ Real-Time PCR System (Thermo Fisher Scientific, Inc.) using QuantiNova SYBR^®^ Green PCR Kit (QIAGEN, Inc.). The thermocycling conditions were as follows: 95°C for 30 sec, followed by 40 cycles of amplification at 95°C for 5 sec, 59°C for 30 sec and 72°C for 30 sec. Relative expression was measured using the 2-ΔΔCq method the expression of U6 was utilized as the internal control for tsRNA. The sequences of the primers used in this study were as follows:

tRF-33-6SXMSL73VL4YDN, 5’-CGTATTCGACGATCGGCCGTGA-3’, and 5’-TACTCTGCGTAGATCGGTTTCCG-3’;
tRF-32-6SXMSL73VL4YK, 5’- CAGCGACGATCGGCCGTGATCGT-3’, and 5’- AGTGGTTAGTACTCTGCG-3’;
tRF-32-M1M3WD8S746D2, 5’-ATCCGTATTCGACGCGGCCC-3’, and 5’-CTCCCGGTGTGGGAA-3’;
tRF-35-RPM830MMUKLY5Z, 5’-CTGCTTGCATGGGTAGCGTGG-3’, and 5’-CGCTGGATTTCGTGCACCG-3’;
tRF-33-K768WP9N1EWJDW, 5’-CGTGCACGCCCCTGGCGGT-3’, and 5’-TTAGGATTCGGCGC-3’;
tRF-32-MIF91SS2P46I3,5’-TATTCGACGCGGCTAGCTC-3’, and 5’- GACTCT CGCAGA-3’
U6-F 5’-CGATACAGAGAAGATTAGCATGGC-3’, and U6-R 5’-AACGCTTCACGAATTTGCGT-3’;

### Small RNA Sequence Processing and Expression Analysis

**Figure 1:**
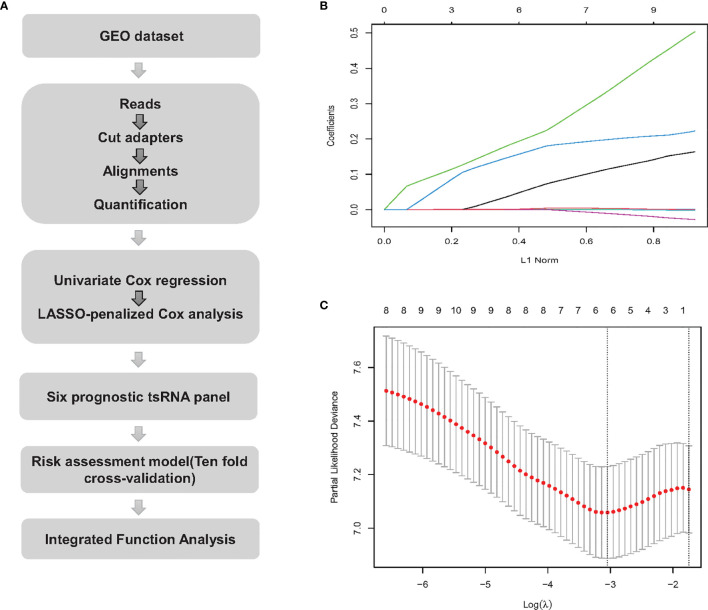
Workflow and LASSO-Cox regression analysis. **(A)** tsRNA expression extraction process and downstream analysis. **(B)** LASSO coefficient profiles of the 10 tsRNAs. The L1 Norm at the optimal value by 1 s.e. criteria return six non-zero coefficients. **(C)** The two dotted vertical lines are drawn at the optimal values by 1 s.e. criteria (left line) and minimum criteria (right line).

All bone biopsies were sequenced on the NextSeq 500 (Illumina) at a final concentration of 2 pM. After quality control, the sequencing fastq reads adapters were trimmed and filtered for ≥ 16 nt using Cutadpt 2.1 (8). Trimmed reads were aligned to mature-tRNA on the entire genome using MINTmap (9). The workflow is shown in **Figure 1A**.

### Statistical Analysis

To identify prognostic biomarkers that predict overall survival, Kaplan–Meier was applied to analyze the correlation between tsRNA and overall survival, and the log-rank test was used to associate survival curves. Multivariate survival analysis was adopted to adjust the risk score and clinicopathological characteristics. All analyses were performed using R software (version 4.0.2).

### LASSO Regression Analysis

To solve the possible over fitting caused by high-dimensional tsRNA expression levels compared to the small number of samples, using a LASSO-regularized linear model is a popular solution (10). Compared to a typical liner model, such as logistic regression, LASSO can use L1 regularization to shrink the coefficient estimates to zero (11). By using ‘penalized’ regression, LASSO can effectively reduce the number of dimensions. We used LASSO regression to eliminate partial tsRNAs. Ten-fold cross-validation was performed to test the linear model and the ‘glmnet’ package in R was used to perform the LASSO regression analysis.

## Results

### Clinical Characteristics of Osteosarcoma Dataset

**Table 1** MGH shows the clinicopathological characteristics of the osteosarcoma dataset. All 80 samples were analyzed through miRNA-seq, and 74 samples remained after filtering by the condition of having at least 5% tumor cellularity. The average age of the patients was relatively low (29.39 years), 49 of 74 (66.2%) were male, and the metastasis rate of patients was 23%. The median follow-up was 98.2 months (SD 0.92), and 28 (37.8%) patients died. **Table 1** TARGET displays the clinical information of 88 RNA-seq osteosarcoma samples. Compared with MGH, the average age (15.17 SD 0.06) of the TARGET sample was lower and the follow-up time (50.8 SD 0.43) was shorter.

### tsRNA Association With the Survival From the Osteosarcoma

**Figure 2:**
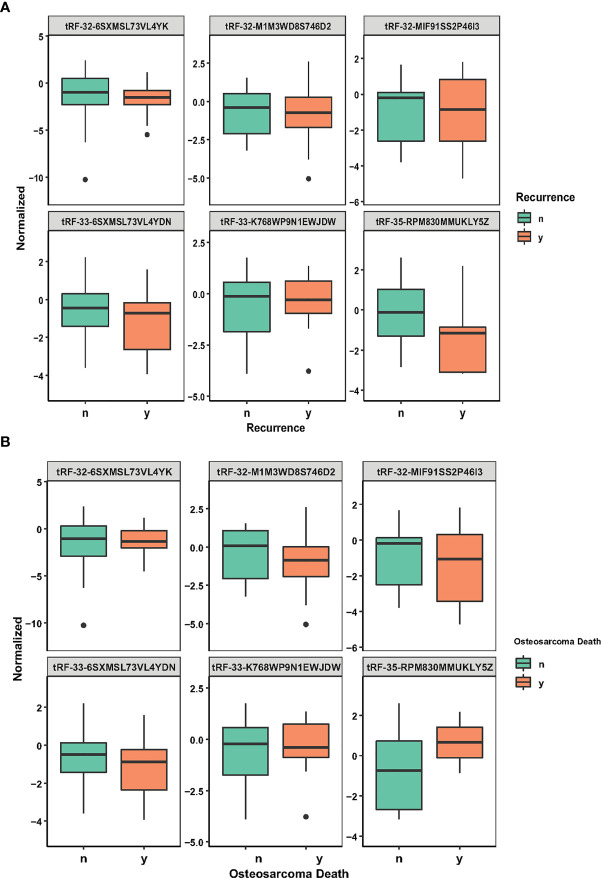
tsRNA expression in recurrence and osteosarcoma death. **(A)** Six LASSO-selected tsRNA expression levels in patients with recurrence and non-recurrence. **(B)** Six LASSO-selected tsRNA expression levels in surviving patients and patients with death from osteosarcoma. The lines from top to bottom are tRF-33-6SXMSL73VL4YDN, tRF-32-6SXMSL73VL4YK, tRF-32-M1M3WD8S746D2, tRF-35-RPM830MMUKLY5Z, tRF-33-K768WP9N1EWJDW, and tRF-32-MIF91SS2P46I3.

**Figure 3:**
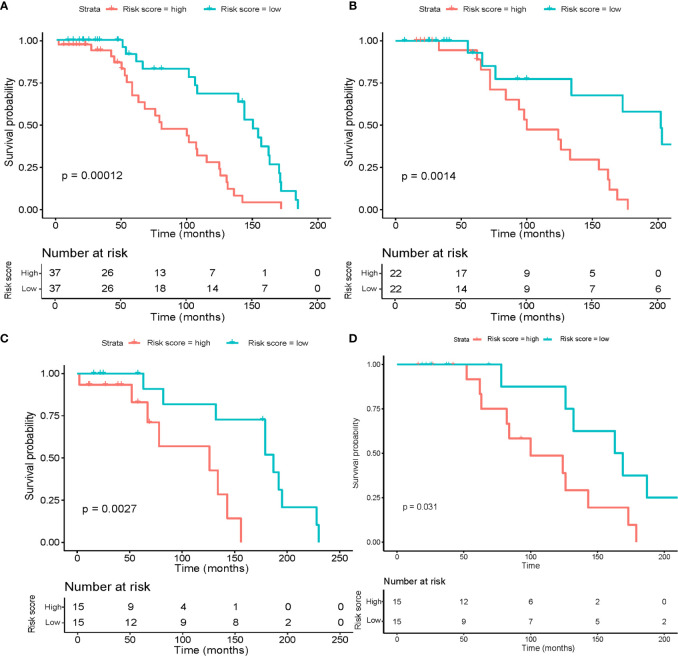
Risk score calculated by the six tsRNA panel and Kaplan–Meier survival analysis results. The OS patients were separated into high-risk and low-risk groups using the median cutoff value. P-values were assessed using the log-rank test. **(A)** Kaplan–Meier survival analysis in the entire dataset. **(B)** Kaplan–Meier survival analysis in the training set. **(C)** Kaplan–Meier survival analysis in the internal validation set. **(D)** Kaplan–Meier survival analysis in the independent validation set.

**Table 2** describes a list of 10 tsRNAs that p-value < 0.05 level with hazard ratio (HR) and 95% confidence interval (CI) when the univariate Cox models were evaluated. We performed a LASSO-Cox regression model (**Figure 1A**) to build a prognostic classifier, and six tsRNAs selected from the 10 tsRNA candidates: tRF-33-6SXMSL73VL4YDN, tRF-32-6SXMSL73VL4YK, tRF-32-M1M3WD8S746D2, tRF-35-RPM830MMUKLY5Z, tRF-33-K768WP9N1EWJDW, and tRF-32-MIF91SS2P46I3. Next, we built the panel using a formula to calculate each patient’s risk score according to their specific six tsRNA expression data (**Figures 1B, C**). We further analyzed these six tsRNAs in patients who relapsed and died from osteosarcoma. Although the expression of some tsRNAs between the two groups shows a trend of separation, no significant differences were observed between them (**Figures 2A, B**). However, LASSO-Cox modeling had an excellent effect on the prognosis analysis of patients with osteosarcoma. Applying the LASSO-Cox regression models, the risk score formula was equal to (0.017*tRF-33-6SXMSL73VL4YDN) + (0.011* tRF-32-6SXMSL73VL4YK) +(2.391*tRF-32-M1M3WD8S746D2) + (0.068*tRF-35-RPM830MMUKLY5Z) + (9.684*tRF-33 K768WP9N1EWJDW) + (7.848*tRF-32-MIF91SS2P46I3). In this equation, the highest risk value was 100 and the lowest risk value was 0. After re-calculating the risk score of every patient, patients with lower risk scores (cutoff = 66.7) typically had better survival rates than those with higher risk scores (**Figure 3A**). The training data set, internal validation (**Figures 3B, C**), and intendent validation set (**Figure 3D**) were applied to verify the model’s predictive ability.

**Table 2:** A list of top 10 tsRNAs with p-value less than 0.05 with hazard ratio (HR) and 95% confidence interval (CI) when the univariate Cox models were assessed.

Name	GtRNAdb names	tRF Sequence	p Values	Hazard Ratio	95% CI
tRF-33-6SXMSL73VL4YDN	tRNA-His-GTG-1-9	GGCCGTGATCGTATAGTGGTTAGTACTCTGCGT	0.00598179	0.4288	0.23-0.799
tRF-32-6SXMSL73VL4YK	tRNA-His-GTG-1-9	GGCCGTGATCGTATAGTGGTTAGTACTCTGCG	0.00861906	0.446	0.24-0.83
tRF-33-K768WP9N1EWJDW	tRNA-Glu-CTC-2-1	CCCCTGGCGGTCTAGTGGTTAGGATTCGGCGCT	0.01088018	0.4466	0.238-0.838
tRF-32-PW5SVP9N15WVN	tRNA-His-GTG-1-9	GCCGTGATCGTATAGTGGTTAGTACTCTGCGT	0.01456028	0.4683	0.252-0.872
tRF-32-MIF91SS2P46I3	tRNA-Lys-CTT-4-1	CGGCTAGCTCAGTCGGTAGAGCATGGGACTCT	0.01852132	0.4947	0.269-0.911
tRF-32-XSXMSL73VL4YK	tRNA-His-GTG-1-9	TGCCGTGATCGTATAGTGGTTAGTACTCTGCG	0.02326819	0.5022	0.275-0.918
tRF-35-RPM830MMUKLY5Z	tRNA-Leu-TAG-1-1	GGTAGCGTGGCCGAGCGGTCTAAGGCGCTGGATTT	0.02560955	0.5133	0.281-0.938
tRF-40-2VR008R959KUMKF6	tRNA-Asp-GTC-3-1	CACGCGGGAGACCGGGGTTCGATTCCCCGACGGGGAGCCA	0.04114561	1.8876	1.022-3.487
tRF-32-M1M3WD8S746D2	tRNA-Glu-TTC-2-2	CGGCCCGGGTTCGACTCCCGGTGTGGGAACCA	0.04215268	0.5412	0.297-0.985
tRF-34-6SXMSL73VL4YHE	tRNA-His-GTG-1-9	GGCCGTGATCGTATAGTGGTTAGTACTCTGCGTT	0.0436662	0.5498	0.302-1.001

### Multivariable Analysis and Ten-Fold Cross-Validation

After multivariable adjustment by clinical variables, the combined tsRNA score model showed excellent predictive power superior to either the tsRNA or clinical score models alone (HR 3.15, 95% CI 0.15–0.66, p<0.001), which was verified in the training (HR 3.44, 95% CI 0.09-0.93, p<0.05) and validation datasets (HR 3.33, 95% CI 0.06-1.41, p<0.05) (**Table 3**). To provide accurate and reliable results, we performed ten-fold cross-validation across the entire dataset (**Supplementary Table S2**). In each fold, the p-value of the risk score in the Kaplan–Meier survival curve was validated.

**Table 3:** Multivariate Cox regression analysis of the risk score with overall survival across the whole dataset.

Parameters	p Values	Hazard ratio	95% CI
Risk score (High)	0.00238	3.1486	0.15-0.67
Age (> 30)	0.02614	0.9792	1.00-1.04
Sex (male)	0.53186	1.2503	0.39-1.61
Recurrence Yes	0.29736	1.5309	0.29-1.45
Disease Grade3/3	0.39023	1.347	0.37-1.46
Disease Grade3/4	0.45715	0.611	0.46-5.99
Disease Grade4/4	0.9011	1.0894	0.23-3.54

### Sequence Matched mRNAs

To predict mRNAs that may interact with the six tsRNAs, we identified a list of mRNAs with p-value less than 0.05 (**Supplementary Table S1**) using univariate Cox analysis. The sequences of distinguished mRNAs were obtained through the UCSC genome browser, and a Percent Identity Matrix was obtained by aligning the sequences of mRNAs with tsRNAs (**Supplementary Figure S1**). We selected the mRNA with the highest matching scores for the corresponding tRNAs: tRF-33-6SXMSL73VL4YDN, tRF-32-6SXMSL73VL4YK with ADCK5; tRF-33-K768WP9N1EWJDW with MYL3; tRF-32-MIF91SS2P46I3 with CTDSP1; tRF-35-RPM830MMUKLY5Z with CTTNBP2NL; and tRF-32-M1M3WD8S746D2 with CMTM1.

### Functional Annotation for Target Genes of Six tsRNAs

**Figure 4:**
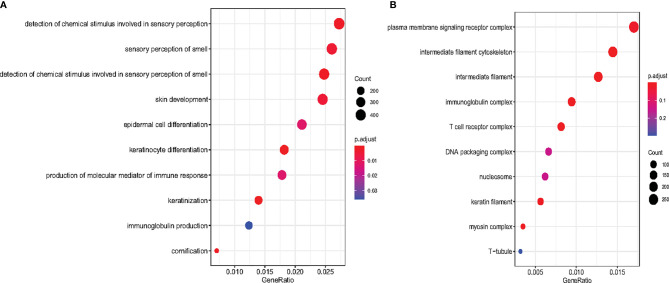
Scatter plot for Biological Process enrichment analysis and Cellular Component enrichment of tsRNA target genes. **(A)** The top 10 Biological Process are presented in the bubble chart. The area of the circle indicates the tsRNA target gene number. **(B)** The top 10 cellular component enrichment are presented in the bubble chart.

To investigate the biological functions of the six tsRNAs, the target genes of the six tsRNA were predicted using RNAhybrid when the tsRNAs had maximum energy less than −25. Furthermore, we carried out the biological process (BP) and cellular component (CC) enrichment analyses for the tsRNA target genes. As shown in the results, the most enriched BP was related to detecting chemical stimuli involved in sensory perception (**Figure 4A**). Several signaling pathways, including sensory perception of smell, skin development, epidermal cell differentiation, and keratinocyte differentiation were affected. In CC enrichment analyses, the most enriched component was related to plasma membrane signaling receptor complex. Intermediate filament cytoskeleton, intermediate filament, and immunoglobulin complex were also significant (**Figure 4B**).

## Discussion

With the rapid development of miRNA sequencing and increasing computing power, the non-coding RNAs (ncRNAs) can now be comprehensively separated and identified. The recent discoveries of various ncRNAs has opened a new way to explain biological control and irregularities. Growing evidence shows that transfer RNA (tsRNA) contributes to biological control and characteristics connected with cancer progression (12). tsRNA has prominent potential as a new cancer diagnosis and prognosis marker, especially the diagnosis of cancer through peripheral blood (13). However, it has not been extensively investigated (5, 14–16).

Osteosarcoma is the most frequent primary malignant bone tumor in adolescents. Before the 1970s, the routine treatment for high-grade OS was amputation (17). Yet despite adjuvant chemotherapy greatly improving patients’ survival rate in the 1970s to 1980s, the diagnosis and treatment techniques have not been renewed for decades. Thus, it is crucial to develop prognostic markers from tsRNA which could help judge therapeutic benefits to osteosarcoma patients. Towards this goal, we have identified a prognostic panel based on tsRNA factors predicting osteosarcoma patients’ overall survival.

In this paper, we have identified a potential tsRNA prognostic panel for osteosarcoma and assessed their risk scores on overall survival, and we provided a new informative pattern to display the impact of the patients’ ncRNAs on overall survival probability. However, using tsRNA is a novel approach for osteosarcoma prognosis, and a clear understanding of tsRNA mechanisms is still lacking. Here, we use sequence matching and RNAhybrid to predict the target genes.

AarF domain containing kinase 5(ADCK5) is a member of an atypical kinase family, which may be involved in transferase activity transferring phosphorus-containing groups as well as protein serine/threonine kinase activity (18). Evidence shows that it is overexpressed in many carcinomas and regulates the expression of tumor oncogene human pituitary tumor transforming gene-1 (PTTG1) to enhance the migration and invasion capabilities of cancer cells (19). Myosin light chain 3 (MYL3) encodes ventricular/cardiac isoform protein, which significantly decreased during fat accumulation in bovine skeletal muscle (20). Carboxy-Terminal Domain RNA Polymerase II Polypeptide A Small Phosphatase 1 (CTDSP1) is related to the drug resistance of colorectal cancers (21). Chemokine-like factor (CKLF)-like MARVEL transmembrane domain-containing 1 (CMTM1) was upregulated in testis and many tumor tissues, and also raised cell proliferation rates and resistance to tumor necrosis factor-α (TNF-α)-induced apoptosis (22). From the biological process (BP) and cellular component (CC) enrichment analyses, the six tsRNA may be involved mainly in detecting chemical stimuli participating in sensory perception and the plasma membrane signaling receptor complex. Although it has not yet been proven that these pathways are closely related to osteosarcoma, these results provide new perspectives for later analysis on these tsRNAs.

There are some limitations to our study. Due to the low incidence of osteosarcoma and resulting paucity of samples for miRNA sequencing, it is difficult to locate adequate miRNA-seq samples for further verification. To the extraction of RNA expression, it can be more convenient by using sRNAtools (23). We will continue to collect specimens in future studies to validate our prognostic markers.

## Data Availability Statement

The original contributions presented in the study are included in the article/**Supplementary Material**. Further inquiries can be directed to the corresponding author.

## Ethics Statement

The studies involving human participants were reviewed and approved by the Fourth Affiliated Hospital of Guangxi Medical University’s ethics committee. The patients/participants provided their written informed consent to participate in this study.

## Author Contributions

ZL had the idea and launched the investigation. ZT collected and processed data. ZT and SZ composed the manuscript. All authors contributed to the article and approved the submitted version. 

## Funding

This work was supported in part by funding from the Key Research and Development Program of Guangxi Zhuang Autonomous Region (No. AB16380152) and in part from the Key Research and Development Program of Liuzhou (2018BJ10509). 

## Conflict of Interest

The authors declare that the research was conducted in the absence of any commercial or financial relationships that could be construed as a potential conflict of interest.

## Publisher’s Note

All claims expressed in this article are solely those of the authors and do not necessarily represent those of their affiliated organizations, or those of the publisher, the editors and the reviewers. Any product that may be evaluated in this article, or claim that may be made by its manufacturer, is not guaranteed or endorsed by the publisher.
