# Supplementary material for: Development of a tRNA-Derived Small RNA Prognostic Panel and Their Potential Functions in Osteosarcoma
Source: Front Oncol. 2021 Aug 2;11:652040. doi: 10.3389/fonc.2021.652040 (PMC8366061; doi:10.3389/fonc.2021.652040)
Supplement: Supplementary file 2 [file DataSheet_2.pdf]

Supplementary Appendix

tRNA-derived Small RNAs and their Potential Functions in Osteosarcoma

Supplementary Table S1. A list of mRNAs that p-value less than 0.05 with hazard ratio (HR) and 95% confidence interval (CI) when the univariate Cox models were assessed.

Name	p Values	Hazard Ratio	95% CI
C1orf198	6.48E-06	0.0952	0.027-0.332
METTL20	5.46E-05	8.335	2.436-28.516
PROSER2	5.52E-05	0.1199	0.035-0.41
PGD	6.60E-05	7.1417	2.342-21.776
USP11	9.23E-05	8.0331	2.331-27.69
MAN2B2	0.000107	8.1792	2.342-28.566
NDNF	0.000116	0.1484	0.049-0.452
REXO2	0.000135	0.1252	0.036-0.437
RNF14	0.000167	6.626	2.158-20.34
TBC1D5	0.000206	7.4339	2.154-25.659
PYCR1	0.000218	0.1577	0.052-0.479
KAZALD1	0.000223	0.1615	0.054-0.486
CNNM4	0.000293	0.16	0.052-0.49
PALB2	0.000295	7.037	2.058-24.066
HARBI1	0.000323	6.1447	2.007-18.811
CGREF1	0.000341	0.1697	0.057-0.509
TTLL1	0.000358	6.118	1.992-18.789
ZFP90	0.000377	6.8653	2.006-23.498
STT3A	0.000388	0.1461	0.043-0.5
RHOV	0.000443	0.1689	0.055-0.517
ERCC4	0.000452	8.8628	2.039-38.515
NCAPD3	0.00047	0.142	0.04-0.499
COA4	0.000512	0.1711	0.056-0.524
XPC	0.000513	5.6832	1.887-17.113
LMO3	0.000521	6.676	1.938-22.995
SPICE1	0.000527	0.1774	0.059-0.533
WDR1	0.000558	6.6127	1.921-22.762
FIG4	0.000577	5.6534	1.865-17.136
HSD11B2	0.000611	0.1733	0.056-0.532
DNAJB2	0.000638	0.1527	0.044-0.529
ST3GAL4	0.000648	0.154	0.045-0.53

FOXRED1	0.000663	0.1557	0.046-0.532
ENGASE	0.00069	0.1726	0.055-0.537
APBB1IP	0.00072	5.4684	1.811-16.516
RPL7A	0.000746	0.1844	0.061-0.554
SNX29	0.000753	5.5638	1.821-17.003
GALNT14	0.000762	0.1852	0.062-0.556
ST3GAL1	0.000815	0.1799	0.059-0.552
PRAME	0.000823	5.4352	1.792-16.482
P4HA3	0.000922	5.4405	1.779-16.638
EI24	0.000967	0.1819	0.059-0.56
ZNF692	0.001003	0.204	0.072-0.577
C2	0.00102	5.257	1.74-15.882
CTF1	0.001064	5.1888	1.729-15.568
RGS9	0.001078	0.1642	0.048-0.565
SYDE2	0.001084	0.1906	0.063-0.577
CTDSP1	0.001108	0.187	0.061-0.573
LINC00482	0.001127	0.1877	0.061-0.573
RASGRP2	0.001187	0.2036	0.071-0.582
FADS2	0.001196	0.1955	0.065-0.586
BIRC2	0.001233	0.1942	0.064-0.587
GPR37	0.001251	0.1901	0.062-0.582
INO80D	0.001303	0.1957	0.065-0.591
FBXL5	0.001307	5.2274	1.707-16.003
FAM76B	0.001311	0.1901	0.062-0.584
TCN2	0.001352	5.038	1.68-15.108
CHEK1	0.001363	0.1953	0.064-0.592
ALDH1A1	0.001379	4.5809	1.652-12.703
HIST3H2A	0.001449	0.2001	0.067-0.6
SH3BGRL	0.001451	4.5347	1.639-12.549
ROBO3	0.001452	0.1901	0.061-0.589
CCDC78	0.001453	0.2136	0.076-0.597
DSEL	0.00146	4.552	1.641-12.625
SRPR	0.001474	0.1991	0.066-0.599
PTH1R	0.001475	0.2271	0.085-0.61
CBL	0.001479	0.1934	0.063-0.593
DUS1L	0.001504	0.1938	0.063-0.595
PRKAR2A	0.001534	5.0169	1.664-15.124
ATMIN	0.001541	4.6524	1.652-13.102
KRT2	0.001557	0.201	0.067-0.604
BUD13	0.00158	0.1948	0.063-0.598
SLC27A3	0.001584	0.2099	0.073-0.601
RNF214	0.001621	0.217	0.077-0.612
TMEM136	0.001634	0.2151	0.076-0.61

MANBA	0.00164	4.4523	1.612-12.297
PNMA1	0.001682	4.5617	1.622-12.832
MDGA1	0.001687	4.9011	1.635-14.695
SLC16A4	0.00169	0.2237	0.081-0.62
PXN	0.001698	4.9371	1.635-14.906
FAM109B	0.001716	0.2032	0.068-0.611
MAPK1IP1L	0.001724	4.5781	1.627-12.88
RPS7	0.001749	0.2176	0.077-0.616
ANKMY1	0.001781	0.2177	0.077-0.616
RPS8	0.001829	0.2198	0.078-0.621
DDX60L	0.001836	4.5258	1.607-12.748
TAF1D	0.001856	0.2183	0.077-0.619
FERMT1	0.00186	0.2202	0.078-0.622
TFF3	0.001884	5.0408	1.639-15.506
CITED2	0.001888	4.8371	1.613-14.509
SLC7A1	0.001933	0.2049	0.068-0.62
NUDT8	0.002011	0.2281	0.082-0.633
BMP2	0.00202	0.2423	0.092-0.64
RAB28	0.002054	4.7951	1.597-14.397
ADD1	0.002064	4.7693	1.592-14.285
MMD	0.002072	4.3314	1.568-11.966
CMIP	0.00211	5.7154	1.639-19.933
GPR133	0.002138	0.229	0.082-0.637
CCDC96	0.002138	4.9726	1.614-15.318
RHBDL2	0.002169	0.2321	0.084-0.641
CLDN6	0.002187	4.0792	1.548-10.75
STRIP2	0.002192	4.421	1.571-12.445
ATM	0.00221	0.2329	0.084-0.643
NXPE3	0.002248	4.4312	1.57-12.508
HS2ST1	0.002258	4.3231	1.558-11.998
PLCB4	0.002277	0.2247	0.079-0.636
SIGLEC17P	0.002305	0.2275	0.081-0.64
HJURP	0.00232	0.213	0.071-0.638
PKIA	0.002331	4.3974	1.56-12.393
FAT3	0.002393	0.2267	0.08-0.641
MKS1	0.002456	4.3647	1.549-12.297
MN1	0.002497	4.6559	1.554-13.947
ABAT	0.002508	4.2609	1.537-11.815
ZNRD1	0.002519	0.2141	0.071-0.643
MTFR1L	0.002527	4.353	1.544-12.27
CYP20A1	0.002559	0.2304	0.082-0.649
USP9X	0.002626	4.2584	1.531-11.846
THUMPD3	0.002638	4.2487	1.529-11.803

GALC	0.002661	4.2087	1.522-11.641
ACTB	0.002685	4.7204	1.548-14.399
ACY3	0.002766	0.2363	0.085-0.657
CSPG5	0.002817	0.2398	0.087-0.663
TRMT44	0.002885	4.269	1.517-12.017
CDCA7	0.00289	0.2171	0.072-0.655
KCNC3	0.002893	4.153	1.504-11.469
PPM1J	0.002954	0.2148	0.071-0.653
FKBP11	0.002996	0.2169	0.072-0.657
MFAP4	0.003007	4.1541	1.5-11.505
IL20RB	0.003031	0.2196	0.073-0.659
MYL3	0.003064	0.2423	0.088-0.669
SLC37A4	0.003092	0.2202	0.073-0.661
FAM188A	0.003112	0.2211	0.074-0.662
TNFRSF10C	0.003177	4.5663	1.51-13.807
SLC8A3	0.003265	0.244	0.088-0.675
TMC5	0.003282	4.5496	1.504-13.762
RPS25	0.003358	0.2384	0.085-0.672
RAD18	0.003415	4.1607	1.483-11.673
RELT	0.003455	0.2412	0.086-0.676
UHRF2	0.003461	0.2238	0.075-0.671
EIF2AK3	0.003472	0.2465	0.089-0.681
TMCC2	0.003485	0.2223	0.074-0.671
SLCO5A1	0.003503	0.225	0.075-0.673
PFKFB3	0.003542	0.2234	0.074-0.674
DDX19A	0.003601	4.08	1.469-11.332
WDR19	0.003708	4.0164	1.456-11.083
PHOSPHO1	0.003754	0.2473	0.089-0.686
CREB3L1	0.003805	0.2492	0.09-0.689
ISCA2	0.003807	4.0205	1.454-11.121
FAM91A1	0.003829	0.225	0.074-0.68
SLC38A4	0.003864	4.0428	1.451-11.264
RPS12	0.003874	0.2501	0.091-0.691
NOG	0.003883	0.2508	0.091-0.692
SLC17A9	0.00389	0.2592	0.097-0.69
LYVE1	0.003902	5.1206	1.489-17.609
CCDC42	0.00396	4.0371	1.447-11.261
FAP	0.003964	4.0948	1.454-11.529
NPC2	0.00403	3.7923	1.435-10.018
CREBZF	0.004117	0.2417	0.085-0.687
WDR63	0.00414	4.0836	1.448-11.519
SORT1	0.004148	4.3991	1.455-13.296
TMEM128	0.004207	4.3906	1.453-13.27

WFS1	0.004231	4.0731	1.444-11.491
DNPH1	0.004251	0.2656	0.101-0.702
RPS3	0.004314	0.2542	0.092-0.701
OPN3	0.004346	0.2532	0.092-0.7
PIKFYVE	0.004376	0.2545	0.092-0.702
ARCN1	0.00438	0.2514	0.09-0.701
JADE2	0.004471	3.9581	1.422-11.017
ADAMTS10	0.00448	0.2537	0.092-0.703
IST1	0.004505	3.9958	1.427-11.189
CPE	0.004553	0.2544	0.092-0.704
SP7	0.004657	0.2564	0.093-0.708
LTK	0.004731	0.2721	0.104-0.714
MYH10	0.004752	3.8835	1.408-10.711
CMTM1	0.004763	4.2639	1.424-12.769
ARL14EP	0.004775	4.2784	1.426-12.84
DZIP1	0.004826	0.2325	0.077-0.702
SLC36A4	0.004889	0.2578	0.093-0.712
RAD21	0.004909	0.2544	0.091-0.71
C14orf180	0.005025	0.2553	0.091-0.713
IRF2BPL	0.005088	3.8687	1.398-10.704
MRPL14	0.005113	0.2756	0.105-0.722
RPL36	0.005174	0.2668	0.099-0.716
TRIP4	0.005252	3.7014	1.388-9.872
TSHZ3	0.005273	3.8411	1.39-10.613
AREL1	0.005316	3.7305	1.391-10.007
HNF4G	0.00532	0.2358	0.078-0.712
MIOS	0.005333	3.6283	1.381-9.536
BSG	0.005337	0.2607	0.094-0.72
RNF41	0.00538	0.2359	0.078-0.713
ADM2	0.005489	0.2779	0.106-0.728
BMP8B	0.005557	0.2619	0.095-0.724
SLC38A9	0.005582	3.6679	1.376-9.779
DHPS	0.005591	0.2622	0.095-0.725
COG3	0.005611	0.2517	0.088-0.716
TXNL4B	0.005641	3.8066	1.377-10.519
EFTUD2	0.005648	3.9787	1.396-11.337
MSANTD2	0.005738	0.2521	0.088-0.718
GPR153	0.005746	0.2641	0.096-0.729
PLA2G15	0.00575	3.8285	1.375-10.663
AMN1	0.005811	3.8295	1.374-10.677
MKRN2	0.005829	3.8582	1.377-10.807
HNRNPC	0.005837	0.2573	0.091-0.725
CNTN1	0.005851	3.7739	1.369-10.407

PDGFD	0.005861	0.2616	0.094-0.729
C12orf5	0.005862	3.84	1.374-10.732
HDLBP	0.005944	0.2799	0.107-0.735
SRP68	0.005945	0.2617	0.094-0.73
ZNF175	0.006003	3.8025	1.366-10.586
FDPS	0.006018	0.2654	0.096-0.733
PER3	0.006023	3.7967	1.365-10.563
BLZF1	0.006034	0.2407	0.08-0.726
DUSP18	0.006042	4.1255	1.377-12.363
ZEB1	0.006115	3.5851	1.357-9.468
TTC9B	0.006128	3.8897	1.374-11.01
MARK2	0.006201	0.2587	0.092-0.73
FARP2	0.006205	0.2665	0.096-0.736
TRABD	0.006261	0.2762	0.104-0.735
C17orf53	0.006265	3.821	1.363-10.713
ID1	0.006282	0.2765	0.104-0.738
CLTCL1	0.006347	3.8261	1.358-10.776
MRPL48	0.006373	0.2417	0.08-0.731
CDH6	0.006381	3.7507	1.355-10.379
UTP23	0.006481	0.2604	0.092-0.735
MAGEA11	0.006522	3.7588	1.35-10.465
NXT2	0.00655	3.7166	1.347-10.251
ATAD2	0.006583	0.2653	0.095-0.741
PTCH1	0.006609	0.2696	0.098-0.743
CPNE5	0.006663	0.2839	0.108-0.747
PCK2	0.006749	0.2467	0.082-0.739
XRCC5	0.006773	0.263	0.093-0.741
FANCF	0.006841	3.693	1.339-10.185
CXXC5	0.006889	3.7017	1.34-10.23
PDE3A	0.006923	3.6942	1.338-10.201
C16orf52	0.007021	4.0335	1.346-12.084
GPRC5B	0.007141	0.2695	0.097-0.75
ASAP3	0.007144	3.8035	1.341-10.79
APCDD1	0.007159	0.2719	0.098-0.751
SQLE	0.007213	0.2699	0.097-0.751
ARHGAP28	0.007253	3.7879	1.34-10.71
STAT5B	0.007286	4.0622	1.342-12.293
HEXDC	0.0073	0.2618	0.092-0.746
NLRP11	0.00731	3.7862	1.338-10.71
ATG9B	0.007376	0.2729	0.099-0.754
MRAS	0.007467	0.2822	0.105-0.755
CYP2R1	0.007544	4.0024	1.333-12.013
ATIC	0.00757	0.2478	0.082-0.75

ZNF689	0.007603	3.5204	1.319-9.396
RAD51C	0.00765	3.675	1.323-10.205
GRAMD1B	0.007654	0.286	0.108-0.758
INPP4A	0.007661	0.2832	0.106-0.756
NDFIP2	0.007699	0.2761	0.1-0.76
KIF25	0.007724	0.2755	0.1-0.76
SSX1	0.007891	3.6616	1.314-10.201
PDE1B	0.007898	3.6256	1.313-10.012
HTRA3	0.0079	3.7252	1.321-10.507
ALDH4A1	0.007918	3.5377	1.316-9.508
ATG4D	0.007922	0.293	0.112-0.767
CARD6	0.007962	3.7201	1.319-10.491
DUSP3	0.008001	3.4445	1.307-9.076
HGF	0.008008	3.6155	1.31-9.98
XCL2	0.00805	3.4222	1.304-8.982
ABHD13	0.008111	0.2735	0.098-0.763
SPCS2	0.008137	0.2777	0.101-0.766
PI4K2A	0.008149	3.6124	1.307-9.981
TNK2	0.008218	0.2882	0.108-0.767
DCTN5	0.008226	3.6566	1.309-10.218
UFD1L	0.00825	3.4997	1.307-9.374
PODN	0.008273	3.5862	1.302-9.877
MON1B	0.008326	3.5919	1.302-9.909
ZNF780B	0.008413	3.9202	1.309-11.736
NOMO1	0.008464	3.4163	1.297-9.001
BMP8A	0.008551	0.2941	0.112-0.773
RASL12	0.008654	0.2895	0.108-0.773
CTTNBP2NL	0.008663	3.3692	1.288-8.813
LOXL4	0.008676	0.2777	0.1-0.772
LTV1	0.008765	3.5688	1.293-9.851
FOXP1	0.008818	3.6681	1.3-10.352
SERPINH1	0.008832	0.28	0.101-0.774
TMEM65	0.008864	0.2924	0.11-0.776
SACS	0.008922	3.3706	1.285-8.843
ZBTB7C	0.008928	0.2809	0.102-0.776
ASNSD1	0.008946	0.2736	0.097-0.772
ANKRD29	0.009155	3.3621	1.28-8.828
LRRC56	0.009206	0.2802	0.101-0.779
SLC4A4	0.009215	0.2991	0.114-0.783
COL22A1	0.009324	0.2999	0.115-0.785
EMC8	0.009331	3.5472	1.282-9.811
CCDC3	0.009368	0.2835	0.103-0.782
NR2F1	0.009445	3.4358	1.281-9.214

CEP57	0.009451	0.2803	0.101-0.781
TRIM17	0.009453	0.2791	0.1-0.78
PSAT1	0.009524	0.2934	0.11-0.783
SLC6A2	0.009532	0.3	0.114-0.786
PLXDC2	0.009576	3.5617	1.278-9.928
SFXN1	0.009602	0.2819	0.101-0.784
GBP1	0.009619	3.3597	1.273-8.867
SIAE	0.009652	0.2834	0.102-0.784
ZIC1	0.009669	3.5164	1.274-9.705
BVES	0.009685	3.3815	1.273-8.985
ADCK5	0.009718	0.3012	0.115-0.789
FZD8	0.009731	0.2841	0.103-0.785
HCRTR1	0.00974	3.3118	1.266-8.661
PHF12	0.009746	3.512	1.273-9.692
AHSA2	0.009811	0.2699	0.094-0.779
GLIPR1	0.009848	3.6048	1.278-10.17
TRIM16L	0.009947	3.3635	1.266-8.933
SOST	0.009967	0.2813	0.101-0.786
TUBB2A	0.009998	3.6821	1.281-10.585

Supplementary Table S2. Risk score calculated by the six tsRNAs panel and Kaplan–Meier survival analysis in ten-fold cross validation.

Fold	Training	Validation
1	2.61E-03	3.43E-03
2	1.29E-03	5.89E-02
3	1.43E-03	2.69E-03
4	1.03E-04	8.88E-03
5	2.45E-02	2.68E-04
6	4.41E-02	4.79E-03
7	1.16E-02	4.91E-02
8	1.58E-02	6.86E-03
9	6.32E-05	3.11E-03
10	7.03E-03	1.75E-02

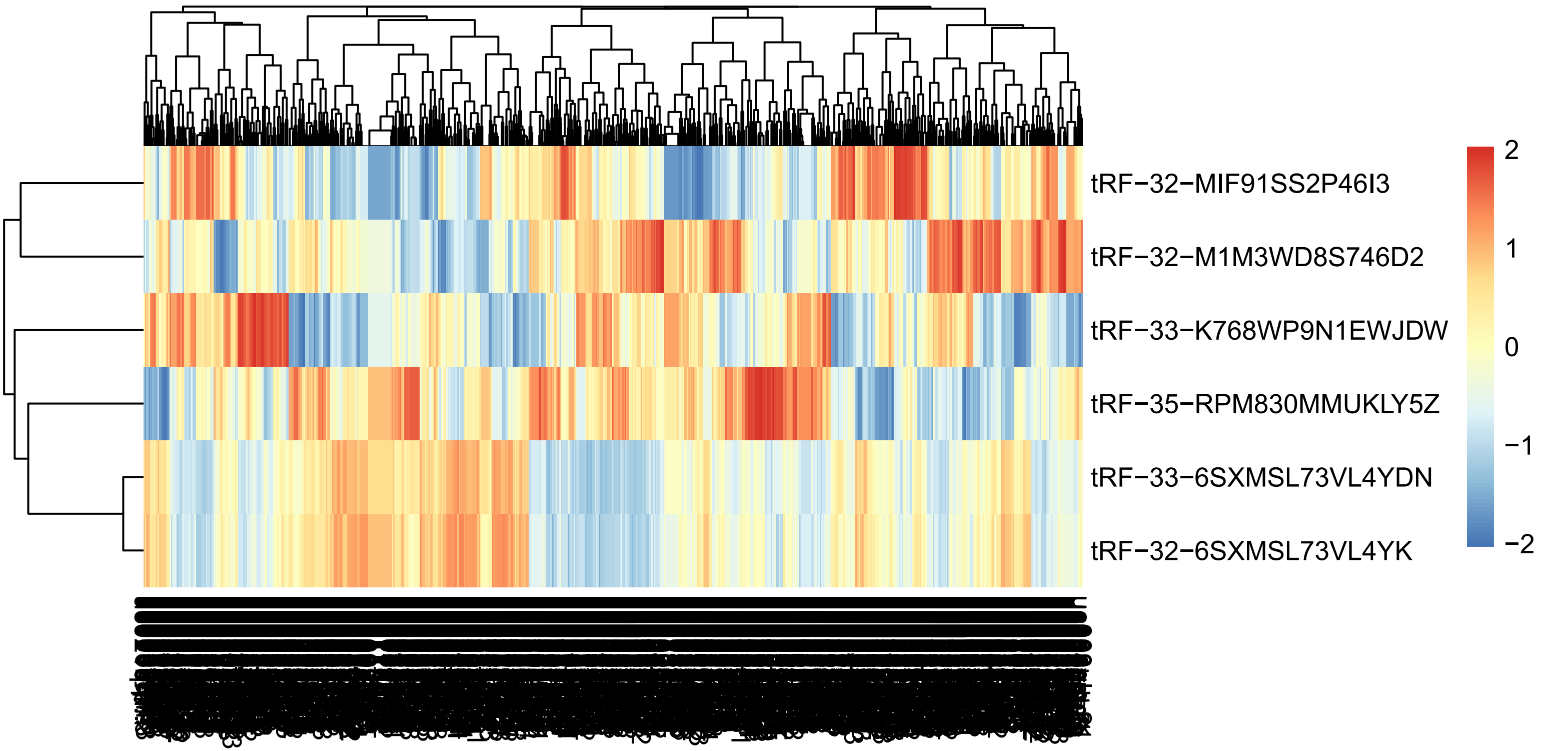

Supplementary Figure S1. Heatmap plot of tsRNA-mRNA sequence match Percent Identity Matrix
